# Resolution of acute inflammation induced by monosodium urate crystals (MSU) through neutrophil extracellular trap-MSU aggregate-mediated negative signaling

**DOI:** 10.1186/s12950-024-00423-9

**Published:** 2024-11-27

**Authors:** Cheng-Hsun Lu, Chieh-Yu Shen, Ko-Jen Li, Cheng-Han Wu, Yu-Hsuan Chen, Yu-Min Kuo, Song-Chou Hsieh, Chia-Li Yu

**Affiliations:** 1 Department of Internal Medicine, National Taiwan University Hospital, No. 7, Chung-Shan South Road, Taipei, 10002 Taiwan; 2 Graduate Institute of Clinical Medicine, National Taiwan University College of Medicine, No.1, Chang-Te Street, Taipei, 10048 Taiwan; 3 Department of Internal Medicine, National Taiwan University Hospital-Hsinchu Biomedical Park, No. 2, Sec. 1, Shengyi Road., Zhubei City, Hsinchu County 302058 Taiwan

**Keywords:** Monosodium urate crystal (MSU), Differentiated HL-60, Neutrophil extracellular trap (NET), NET area, NET-MSU aggregate, Estimate inflammation score, Intracellular cytokine signaling regulator

## Abstract

**Background:**

Polymorphonuclear neutrophils (PMN) activation by monosodium urate crystals (MSU) is crucial to acute gouty arthritis and subsequent spontaneous remission within 7–10 days. Activated PMNs release neutrophil extracellular traps (NETs) that entrap MSU crystals, forming NET-MSU aggregates. Whether NET-MSU aggregates contribute to the resolution of acute inflammation remains to be elucidated. This study uses a cell-based approach to unveil their molecular bases.

**Methods:**

All-trans retinoic acid-differentiated HL-60 cells (dHL-60) served as surrogate PMNs. NET release from MSU-activated dHL-60 was confirmed by detecting DNA, neutrophil elastase, and citrullinated histone 3, forming large NET-MSU aggregates. NET area was measured with Fiji software after SYTOX Green staining. Released pro-inflammatory cytokines IL-8 and TNF-α, and the anti-inflammatory cytokine IL-1RA in culture supernatants were quantified to calculate the estimate inflammation score (EIS). Cellular redox state was determined by a FRET-based sensor. Expression of intracellular positive (ERK1/2) and negative (SHP-1 and SHIP-1) cytokine signaling regulators was detected by western blot. qPCR detected mRNA expressions of CISH and SOCS1–SOCS7. Flow cytometry measured neutrophil N1 (CD54) and N2 (CD182) surface markers after staining with fluorescent-conjugated antibodies.

**Results:**

Incubating dHL-60 with MSU for 4 h maximized NET-MSU aggregate formation and acute inflammation with an EIS of 11.6. Prolonging the incubation of dHL-60 + MSU to 22 h gradually raised the EIS to 19.40 without increasing NET area, due to reduced cellular redox capacity. Adding both new dHL-60 and new MSU crystals to the culture, mimicking the clinical scenario, increased NET area but conversely suppressed EIS to 1.53, indicating acute inflammation resolution. The resolution of acute inflammation following prolonged incubation was attributed to decreases in P-ERK and increases in P-SHP-1, SOCS2, SOCS3, and CISH gene expressions, which may suppress pro-inflammatory and enhance anti-inflammatory cytokine production. Moreover, the large NET-MSU aggregates facilitated N1 to N2 polarization, crucial for accelerating inflammation resolution.

**Conclusion:**

We explored the potential molecular basis for the spontaneous resolution of MSU induced acute inflammation using a cell-based model in that huge NET-MSU aggregates frustrate the transformation of newly entering PMNs to the N2 phenotype, enhancing the production of the anti-inflammatory cytokine IL-1RA.

**Supplementary Information:**

The online version contains supplementary material available at 10.1186/s12950-024-00423-9.

## Background

Gouty arthritis (GA) is an episodic acute mono-arthritis triggered by monosodium urate crystals (MSU) in the synovial joints with spontaneous resolution in 7–10 days [1, 2]. It is quite interesting that the rapid fluctuation of serum uric acid levels can also induce acute gout attack. Conversely, the gout patients can also maintain a long-term asymptomatic period between flares (inter-critical gout) despite of hyperuricemia [3–5]. These paradoxical observations may suggest the possibility of MSU surface-coated proteins involving in modulating the inflammation dynamics [6–8]. TNF-α, IL-8, and IL-1β are the key inflammatory cytokines in gouty arthritis [9, 10]. The spontaneous acute GA resolution has been reported association with increased production of anti-inflammatory cytokines including TGF-β1, IL-10, soluble TNF-α receptor and IL-1 receptor antagonist (IL-1RA) from mononuclear cells [11]. In addition, the clearance of the infiltrated inflammatory cells by non-inflammatory phagocytes had been also suggested [12–15].

Polymorphonuclear neutrophils (PMN) are the most prominent inflammatory cells in the synovial fluid during acute gout attack [16]. Many authors have reported that MSU crystals could activate PMNs release of neutrophil extracellular traps (NETs) for NET-MSU aggregate formation [17, 18]. In general, the process of NET release is referred as NETosis. Schauer et al. [19] reported that impaired NETosis exacerbated gout inflammation in mouse experiments. Besides, the proteolytic enzymes attached in NETs could limit inflammation by degrading pro-inflammatory cytokines and chemokines in the acute gout milieu [19, 20]. Interestingly, the amount of NETs in acute gout inflammation depends on the number of MSU crystals rather than leukocytes per se [21]. It is suggested that the entrapment of MSU crystals by NETs can form the large NET-MSU aggregates to modulate acute gout inflammation [22, 23]. Steiger et al. [12] firstly proposed the concept of non-inflammatory NETosis in GA. However, the molecular basis underlying spontaneous acute gout inflammation resolution remains to be explored. Recently, PMNs have been classified into pro-inflammatory N1 and anti-inflammatory N2 phenotypes [24]. The dynamic change of N1 to N2 phenotype in spontaneous acute GA resolution also merits further elucidation.

We hypothesize that the NET-MSU aggregates contribute to acute gout resolution by promoting a shift of newly recruited PMNs into the gout joint towards an anti-inflammatory N2 phenotype. In the present study, we utilized all-trans retinoic acid (ATRA)-differentiated HL-60 cells as surrogate PMNs for investigating the role and the molecular bases of NET-MSU aggregates in spontaneous acute gout resolution.

## Methods

### Differentiation of HL-60 promyeloblast cell line into granulocyte-like cells by ATRA

The HL-60 promyeloblast cell line (CCL240, ATCC, Rockville, MD, USA) was grown in RPMI-1640 containing 10% heat-inactivated FBS and antibiotics at 37 °C in 5% CO_2_-95% air [25]. The HL-60 cells were differentiated to granulocyte-like cells by 10 µM ATRA (Sigma-Aldrich, St. Louis, MO, USA) and 25 ng/mL G-CSF (PeproTech, Rocky Hill, NJ, USA) in 24-well plates for 5 days [26]. The differentiated HL-60 cells (dHL-60) were confirmed by staining with PerCP-Cy5.5-anti-CD11b antibody (BD Biosciences, UK) as described in our previous report [27].

### Identification of NET-MSU aggregate formation by different immunofluorescence antibody staining

For simulating the low protein content in the pre-attack joint fluid, the dHL-60 cells were previously cultured in 2% FBS in RPMI-1640 for 1 h before interaction with MSU crystals. The dHL-60 cells (1.5 × 10^6^ cells/mL) were then cultured on 12-well chamber slides pre-coated with 0.01% L-lysine (Ibidi, Gräfelfing, Germany). Commercially available MSU crystals (InvivoGen, San Diego, CA, USA) are firstly diluted in RPMI (5 mg/ml) to form a uniform suspension after pipetting. Subsequently, they are further diluted in the medium to the required concentration according to experimental conditions. After interaction with MSU crystals from 12.5 to 400 µg/mL for 1–22 h, the NET-entrapped MSU crystals were firstly confirmed by their needle-shaped microcrystals with negative birefringence under compensatory polarized light microscopic observation (CPLM). The NET components in the aggregates were then identified by stain with DAPI (1:10,000, Invitrogen, Carlsbad, CA, USA) for DNA, Alexa Fluor 488-conjugated anti-neutrophil elastase (anti-NE) antibody (1:100, Paso Robles, California, USA), and rabbit anti-citrullinated histone H3 (CitH3, citrulline R2 + R8 + R17, 1:1,000, Abcam, Cambridge, MA, USA) overnight, followed by Alexa Fluor 594-conjugated goat anti-rabbit antibody (1:1,000, Invitrogen, Carlsbad, CA, USA) as secondary antibodies. After washing, the NET-MSU aggregates were observed under a fluorescence microscope.

### Quantitation of NET area by SYTOX green stain after dHL-60 and MSU crystal interaction for 1–22 h

The viable dHL-60 cells were previously labeled with 1 µM CellTracker™ Red CMTPX probe (Thermo Fisher Scientific, Waltham, MA, USA) at 37 °C for 30 min. The stained cells (3.0 × 10^5^ cells/0.2 mL) were then interaction with MSU crystals in micro-wells for 1–22 h. SYTOX Green (250 nM) stain was used for NET area measurement. The △NET area denoting the velocity of NET formation was also calculated. Time-lapse imaging (ImageXpress, Molecular Devices, CA, USA) was used for dynamically capturing the dHL-60-MSU interactions. The areas with green fluorescence (three or nine fields per well at 10X or 20X magnification) were analyzed with Fiji software for measuring the NET area (mm^3^/field or % field per well) [28–30].

### Quantitation of pro-inflammatory IL-8 and TNF-α and anti-inflammatory IL-1RA cytokines in the culture supernatants for calculating the estimate inflammation score (EIS)

dHL-60 cells (3.0 × 10^5^ cells/0.2 mL) were incubated with or without MSU (200 µg/mL) for various hours. The amounts of IL-8, TNF-α, and IL-1RA in the culture supernatants were measured by the respective ELISA kit (R&D Systems, Minneapolis, MN, USA) according to the manufacturer’s instructions. The detection limit is 7.5 pg/mL for IL-8, 6.23 pg/mL for TNF-α, and 18.3 pg/mL for IL-1RA.

Estimate Inflammation Score (EIS) was developed as a simplified metric to estimate changes in inflammation [31, 32]. In this cell-based study, EIS was calculated by the following equation:

$${\rm{EIS = }}\left\{ {{{{\rm{IL - 8}}\left( {\rm{T}} \right)} \over {{\rm{IL - 8}}\left( {\rm{C}} \right)}} + {{{\rm{TNF - \alpha }}\left( {\rm{T}} \right)} \over {{\rm{TNF - \alpha }}\left( {\rm{C}} \right)}} - {{{\rm{IL - 1RA}}\left( {\rm{T}} \right)} \over {{\rm{IL - 1RA}}\left( {\rm{C}} \right)}}} \right\}\,\,{\rm{where}},$$

T (treatment): the mean production of cytokine by dHL-60 + MSU crystals (200 µg/mL).

C (control): the mean production of the same cytokine by dHL-60 + medium.

### Disruption of DNA scaffold in the NET-MSU aggregates by treatment with DNase I

To disrupt the DNA scaffold in the NET-MSU aggregates, recombinant human DNase I (30 IU/mL, Abcam, Cambridge, MA, USA) was simultaneously added to the mixture of MSU (200 µg/mL) and dHL-60 (3.0 × 10^5^ cells/0.2 mL), followed by incubation at 37 °C for 4–8 h. The structure changes of NET-MSU aggregates were observed and NET areas were measured after SYTOX Green stain (250nM, Invitrogen, Carlsbad, CA, USA) as described above.

### Addition of new MSU and new dHL-60 into the 4 h-incubated dHL-60 + MSU mixture for another 18 h incubation mimicking the clinical scenario

After 4 h of incubation, the old supernatant was replaced with the same amount of new MSU and dHL-60 cells for an additional 18 h of incubation. After incubation, both NET area and the amount of pro-inflammatory and anti-inflammatory cytokines were measured for calculating EIS as described in the above paragraph.

### Detection of the cellular oxidation-reduction (redox) state

The dHL-60 cells were previously stained with a FRET-based redox sensor (10µM, StressMarq Biosciences, Victoria, Canada) and were incubated with or without MSU (200 µg/mL) in micro-wells (3.0 × 10^5^ cells/0.2 mL) for 4 h. The cellular redox state was monitored by a high-content confocal system (ImageXpress, Molecular Devices, CA, USA) in that blue fluorescence reflects redox reductive, whereas the green fluorescence denotes redox oxidative state in the cells. Nine images per well were analyzed by Fiji software for the average blue/green fluorescence intensity ratio. For more contrast, the SYTOX Red (2 nM, Invitrogen, Carlsbad, CA, USA) instead of SYTOX Green, was used for staining the NET area.

### Detection of phosphorylated intracellular cytokine signaling regulators by Western blot

dHL-60 cells (3.0 × 10^5^ cells/0.2 mL) were incubated with or without MSU (200 µg/mL) for 2–4 h followed by lysis in RIPA buffer containing protease inhibitor cocktail (Roche, Penzberg, Germany). The cell lysates were electrophoresed in 10% SDS-PAGE and were then transferred to PVDF membranes (Millipore, Billerica, MA, USA) and were probed by rabbit antibodies against ERK1/2, phospho-ERK1/2 (Thr202/Tyr204), SHP-1, phospho-SHP-1 (Tyr564), SHIP1, or phospho-SHIP1 (Tyr1020) (Cell Signaling Technology, Danvers, MA, USA), followed by HRP-conjugated anti-rabbit IgGs. After washes, the relative protein amounts were analyzed by densitometry.

### Quantitation of the intracellular CISH and SOCSs mRNA expression by RT-PCR

Total RNAs were extracted from dHL-60 cells ± MSU after 1–4 h incubation by using TRIzol kit (Invitrogen, Carlsbad, CA, USA). These RNAs were then reversely transcribed to cDNA using iScript™ cDNA synthesis kit (Bio-Rad, Berkeley, CA, USA). qPCR was performed by a QuantStudio System (Applied Biosystems, Foster City, CA, USA) for 40 cycles in duplicate. The mRNA amounts were normalized to cyclophilin A. The primer pair sequences are shown in Table S1.

### Identification of N1/N2 phenotype by flow cytometry after stain with surface marker CD54 for N1 and CD182 for N2

dHL-60 cells (3.0 × 10^5^ cells/0.2 mL) ± MSU crystals (200 µg/mL) were incubated for 0.5–2 h. The MSU crystals were dissolved and the cells were concomitantly fixed in 4% formaldehyde. The fixed cells were washed with PBS, and then were stained with BV421-conjugated anti-CD54 (for N1), PE-conjugated anti-CD182 (for N2), PerCP-Cy5.5-conjugated anti-CD11b, and fixable viability dye eFluor™ 780 (BD Biosciences, Oxford, UK). The stained cells were analyzed by a Cytek Aurora flow cytometer (Cytek Biosciences, Fremont, CA, USA) for measuring the median fluorescence intensity of N1 and N2 polarized cells.

### Statistical analysis

Data were statistically analyzed by Mann–Whitney U test using GraphPad Prism 8.0.2 (GraphPad Software Inc. San Diego, CA, USA). The data are displayed in bar graphs as means ± SEM. All experiments were repeated for more than three times. Statistical significance was defined as *p* < 0.05.

## Results

### Identification of dHL-60 cells as mature PMNs after ATRA-induction

**Fig. 1:**
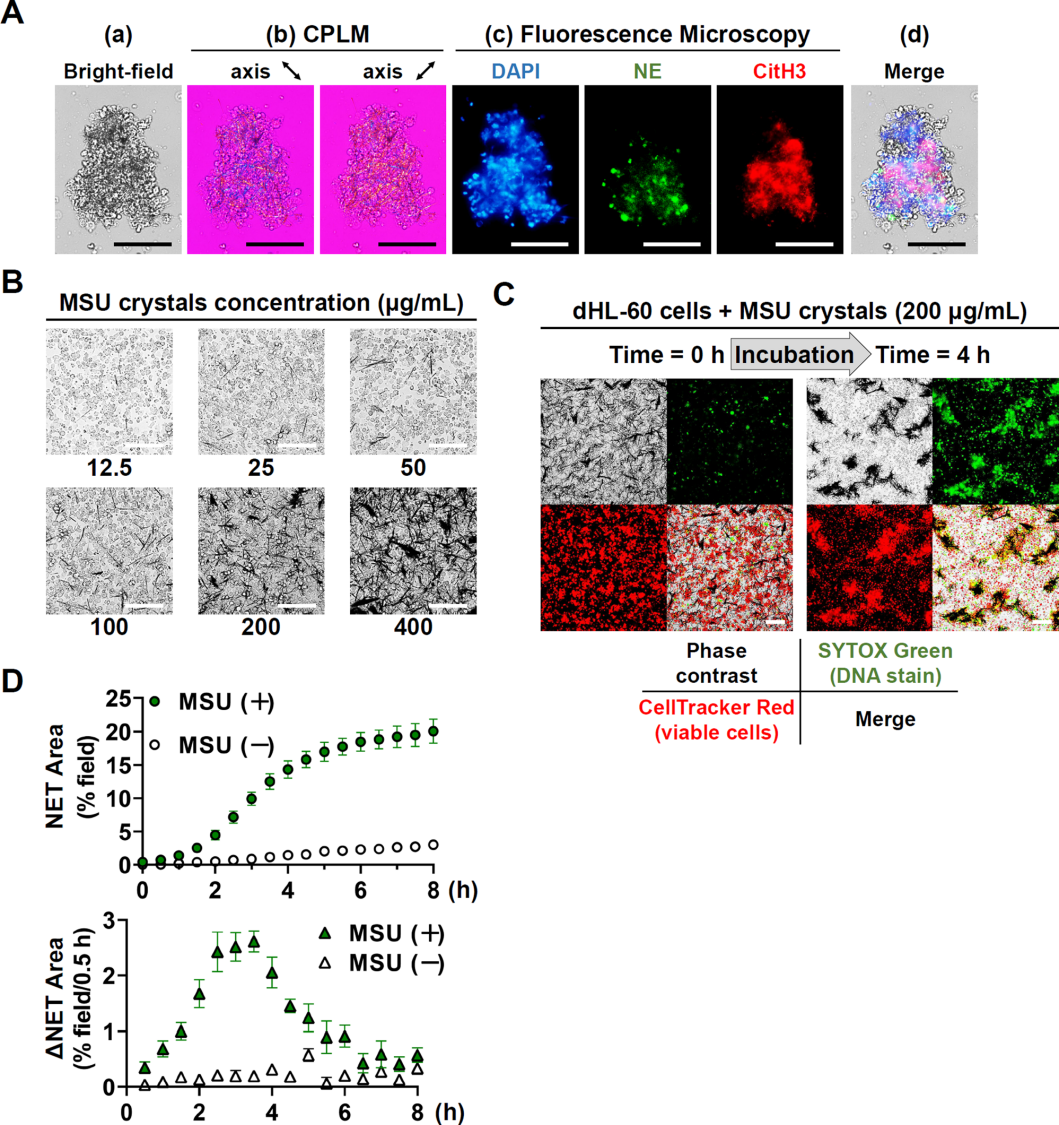
Dose-response and kinetic formation of NET-MSU aggregates on the NET area. (**A**) Representative images show negatively birefringent needle-shaped monosodium urate crystals entrapped in NETs, composed of DAPI-stained DNA, neutrophil elastase, and citrullinated histone 3. (**B**) Dose-responsive effects of MSU from 12.5 to 400 µg/mL on stimulating dHL-60 NET formation under phase-contrast microscopic observation. (**C**) Effect of MSU 200 µg/mL on viable dHL-60 cell (positive CellTracker Red stain) release of SYTOX Green (+) DNA (NETs) for evaluating NET area. (**D**) Kinetic measurement of NET area after SYTOX Green staining of the MSU-activated dHL-60 NET formation for 0–8 h (upper part). We also calculated the NET forming velocity (△NET) kinetically after incubation of dHL-60 ± MSU for 0–8 h (lower part). Data are shown as mean ± SEM of five experiments. Scale bars = 100 µM

Not only did > 90% of ATRA-stimulated dHL-60 cells express CD11b (Fig. S1), they also released neutrophil elastase (NE) associated with NET structures [Fig. 1A-(c)]. Additionally, these cells exhibited CD182 expression (Fig. 7). Obviously, the dHL-60 can be considered as mature PMNs.

### Confirmation of NET-MSU aggregate formation after dHL-60 and MSU crystals interaction

A huge NET-MSU aggregate was found under light microscope after dHL-60 and MSU interaction for 4 h [Fig. 1A-(a)]. The aggregate showed negative birefringence with needle-shaped crystals under CPLM [Fig. 1A-(b)]. The aggregate was found containing DNA confirmed by stain with DAPI, NE and citrullinated histone 3 (CitH3) under fluorescence microscopic observation [Fig. 1A-(c)]. The merged photo of NET-MSU aggregate is shown in Fig. 1A-(d).

### Dose-response and kinetic formation of NET-MSU aggregate for measuring NET area and EIS

**Fig. 2:**
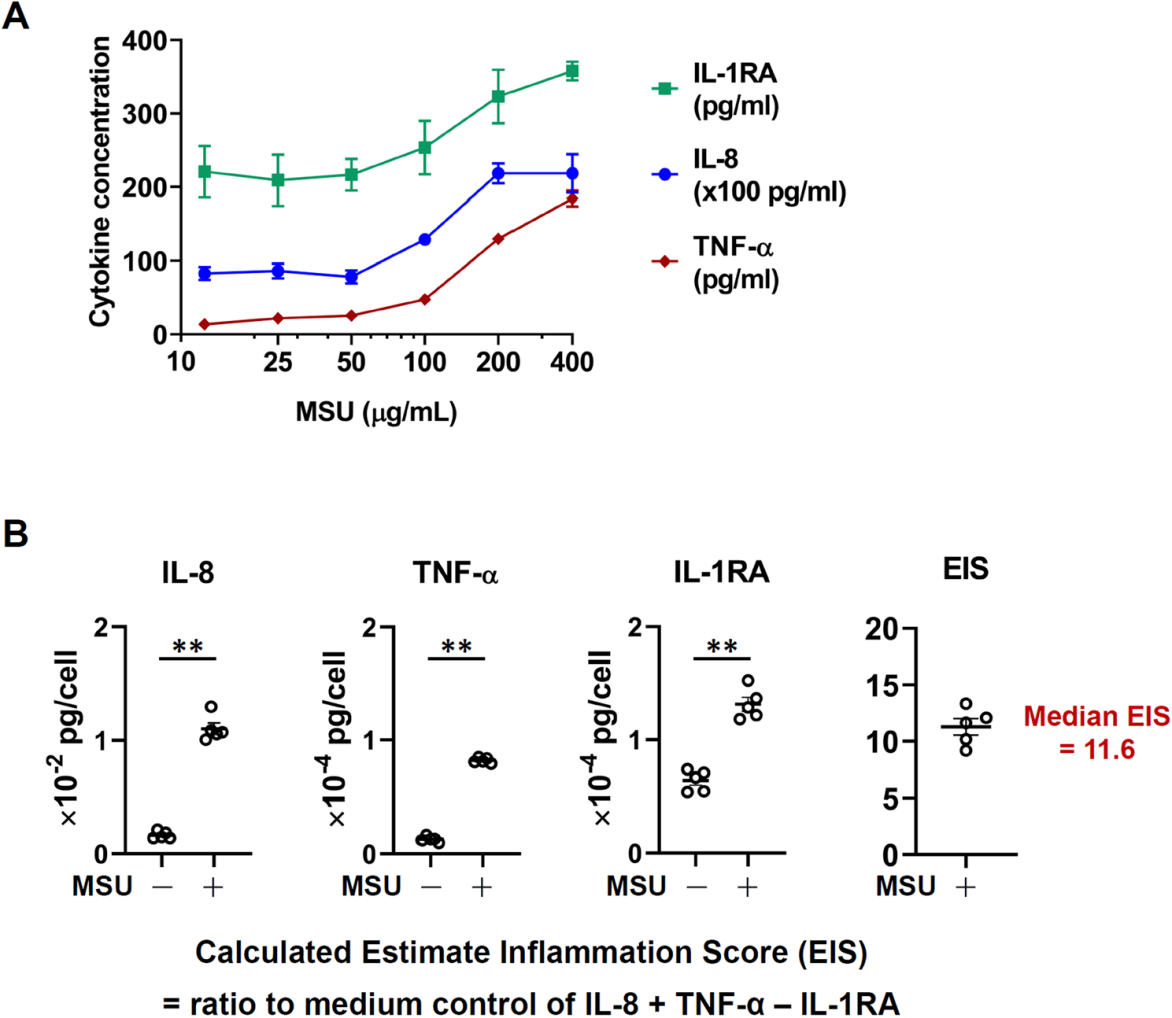
Production of pro-inflammatory and anti-inflammatory cytokines by 4 h incubation of dHL-60 ± MSU for calculation of EIS. (**A**) Dose-responsive effects of MSU (10–400 µg/mL) on dHL-60 cell production of pro-inflammatory (IL-8 and TNF-α) and anti-inflammatory (IL-1RA) cytokine in 4 h incubation. (**B**) The EIS was calculated as the ratio of pro-inflammatory (IL-8 + TNF-α) and anti-inflammatory (IL-1RA) cytokines after incubation with MSU (200 µg/mL) or medium. The EIS reached 11.6 (acute inflammation) in 4 h incubation. Data are shown as mean ± SEM of five experiments. **p < 0.01 by Mann-Whitney U test

Firstly, we confirmed that 200 µg/mL of MSU are the optimal concentration for activating NET-MSU aggregate formation after 2–4 h incubation under light microscope (Fig. 1B). The positive SYTOX Green stain denotes NET formation and can be used for NET area measurement (Fig. 1C- right upper panels stained with SYTOX Green) under fluorescent microscope that are made by the viable dHL-60 cells (Fig. 1C- left lower panels after staining with Cell Tracker Red). The NET area formation reached plateau after 6 h interaction (Fig. 1D, upper part) whereas the velocity of NET formation, △NET area, decrease after 4 h interaction (Fig. 1D-lower part and Supplementary Video S1). Besides, the pro-inflammatory cytokine IL-8 production reached maximum by 200 µg/mL MSU stimulation for 4 h (Fig. 2A). Accordingly, we decided MSU 200 mg/mL as optimal concentration for activating dHL-60 cells for 4 h (acute inflammation). At this time point, we measured the amount of pro-inflammatory cytokine IL-8 and TNF-α, and anti-inflammatory cytokine IL-1RA for calculating EIS. It revealed that the calculated EIS was 11.6 after 4 h incubation as shown in Fig. 2B.

### Effects of disruption of DNA scaffold structure in NET-MSU aggregates by DNase I on NET area and EIS

**Fig. 3:**
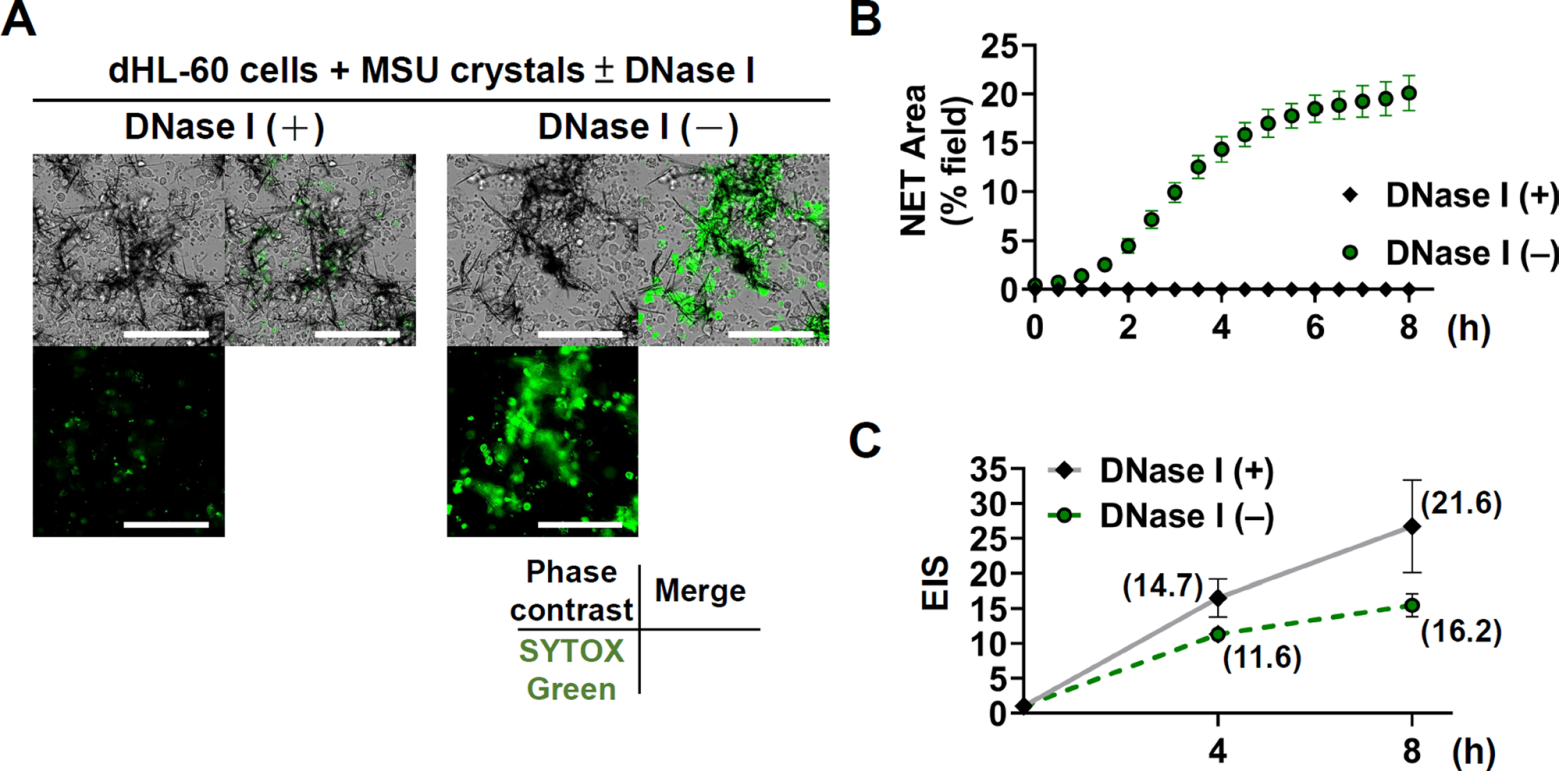
The effects of DNA scaffold structure disruption in NET by DNase I. (**A**) Compared to DNase I (-), DNase I-treated NET-MSU aggregates show dramatically reduced SYTOX Green staining, as illustrated in the florescence microscopy images (left lower parts) and the merged photos (right upper parts) of the representative images. (**B**) The NET area became zero after DNase I- treatment for 8 h. (**C**) Comparison of EIS between DNase (+) and DNase (-)-treated dHL-60 + MSU mixture for 8 h in that higher EIS was noted in DNase (+) group. Data are shown as mean ± SEM of 5 or 6 experiments. Scale bars = 100 µM

Since the velocity of NETs area formation (△NETs area) gradually decelerated after 4 h incubation (Fig. 1D, lower part), we speculate that certain negative control mechanisms would be initiated by NET-MSU aggregates for suppressing dHL-60 cell activation. For confirming this, we then disrupted the DNA scaffold structure in NET-MSU aggregates by DNase I digestion compared to control (Fig. 3A and B). Obviously, SYTOX Green stain representing the DNA scaffold structure was remarkably diminished after DNase I digestion (Fig. 3A and B). Unexpectedly, The EIS conversely increased from 11.6 to 14.7 after 4 h incubation and from 16.2 to 21.6 after 8 h incubation by DNase I (+) compared to DNase (-) as shown in Fig. 3C. It is speculated that the small DNA fragments after DNase I digestion can become danger molecules to activate pattern recognition receptors on dHL-60 cell surface for inducing inflammation [33]. In contrast, the large intact DNA web structure can serve as physical barrier for further NET formation and provides certain anti-inflammatory mechanisms leading to the EIS slowly increase from 11.6 to 16.2 after 8 h incubation (Fig. 3C). For further elucidating the molecular basis of NET-MSU aggregates on suppressing acute inflammation, we prolonged the incubation period from 4 h to 22 h with addition of half amounts of new MSU and new dHL-60 cells as in clinical situation for further observing the changes of NET area and EIS.

### Dynamic changes of NET area and EIS after prolong incubation from 4 h to 22 h with addition of half new MSU and new dHL-60 cells

**Fig. 4:**
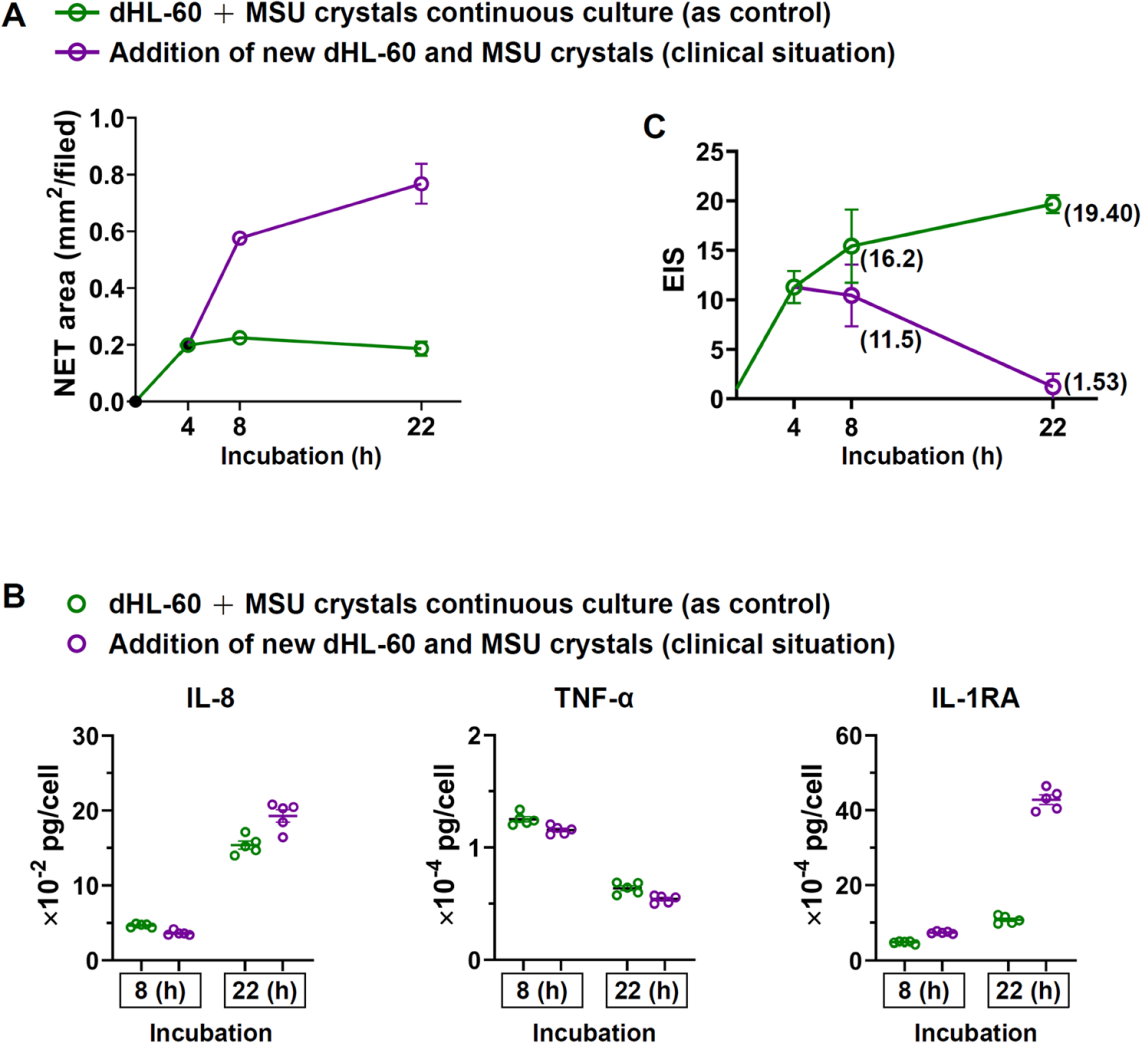
The changes of NET area and EIS after prolonged incubation of dHL-60 + MSU for 22 h. (**A**) Comparisons of NET area between dHL-60 + MSU for 22 h continuous culture (as control) vs. the addition of new dHL-60 and new MSU at 4 h incubation and continuous for another 18 h culture (as clinical scenario). (**B**) Comparison of pro-inflammatory IL-8 and TNF-α, and anti-inflammatory IL-1RA cytokines production between control and clinical scenario group. (**C**) Comparison of EIS between the two groups. The numbers in the graphs denote EIS. Data are shown as mean ± SEM of five experiments

In the continuous culture of old dHL-60 cells with old MSU, the NET area showed no significant extension from 4 to 22 h (*p* = 0.730, Fig. 4A), while the EIS continuously increased from 11.6 at 4 h to 19.4 at 22 h (*p* = 0.016, Fig. 4C). We have shown that the maximal NET formation of MSU-activated dHL-60 cells is between 2 and 6 h in continuous incubation (Fig. 1D). After prolonged incubation to 22 h, the previously engaged dHL-60 cells would stop NET formation after encountering the huge NET-MSU aggregates whereas these cells continuously produced less amount of the pro-inflammatory cytokines. As a result, the EIS gradually accumulated to 19.40 but NET area remained stationary. On the other hand, addition of new MSU and new dHL-60 potently increase in NET area (Fig. 4A) but conversely decrease in EIS from 11.6 (4 h) to 11.5 (8 h) and finally to 1.53 (22 h) (Fig. 4C) due to dramatic increase of IL-1RA (Fig. 4B). It seems that the newly added dHL-60 changed their cytokine profile to increased anti-inflammatory IL-1RA production.

### Cellular redox changes in 4-h incubation of dHL-60 with MSU crystals

**Fig. 5:**
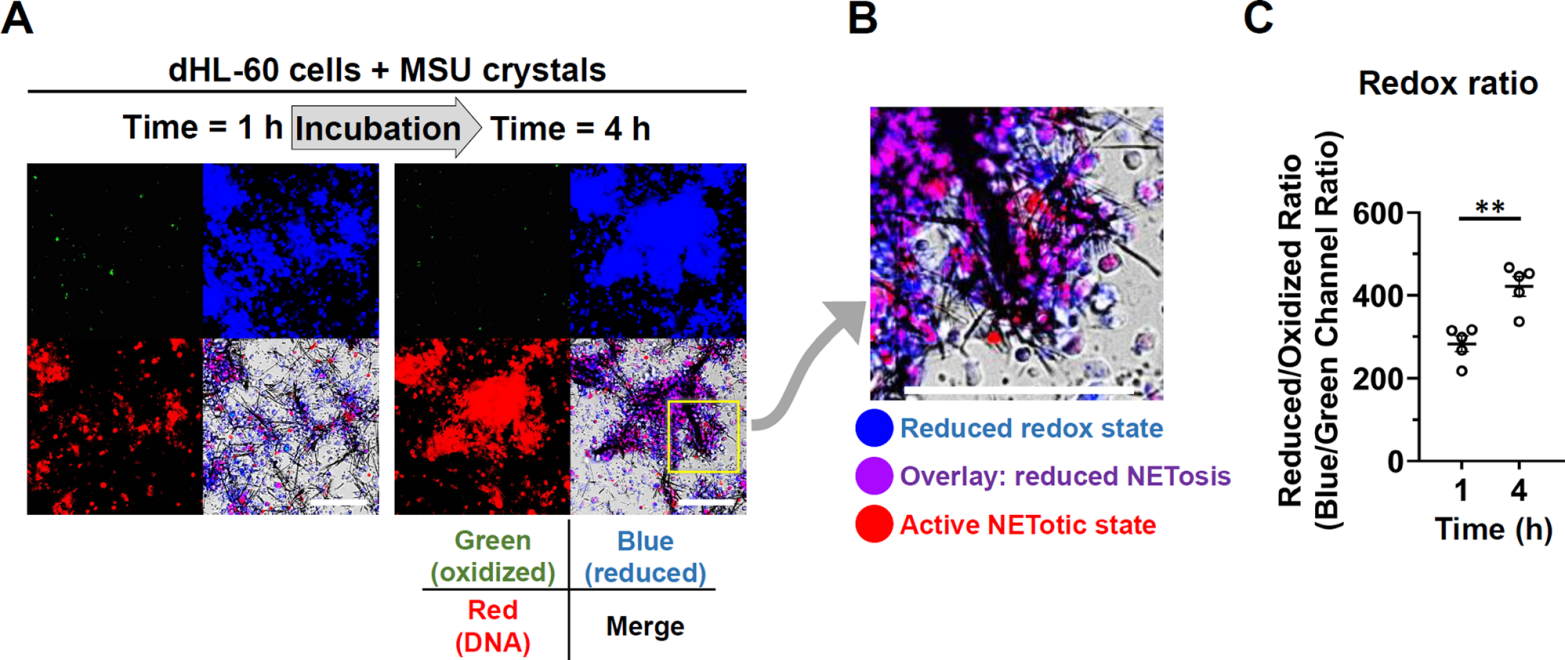
Kinetic changes of cellular redox state in dHL-60 + MSU for 4 h incubation. (**A**) Representative images showing the cellular redox state. The green color represents cells with oxidized state whereas the blue color represents cells with reductive state after 1 h and 4 h incubation. (**B**) Most of the cells entrapped in or around the NET-MSU aggregates shows purple color with reduced NETotic capacity. Only a few cells remain red color in active NETotic state. (**C**) Comparison of reductive/oxidative ratio in dHL-60 cells after 1 h and 4 h incubation with MSU crystals. All experiments were performed five times. Data are shown as mean ± SEM. **p < 0.01 by Mann-Whitney U test. Scale bars = 100 µM

For investigating the molecular basis of decelerated NET formation after 4-h incubation, the cellular redox state was observed since NETosis is highly dependent on high cellular oxidative state. We found that the cellular redox state was gradually shifted to reductive state (blue fluorescence) from 1 to 4 h incubation (Fig. 5A). As shown in Fig. 5B, the purplish-colored cells with cellular reductive redox and decreased NETotic capacity were dominant than the red-colored cells with normal NETotic capacity. Furthermore, less number of oxidized cells with green fluorescence stain was observed around the NET-MSU aggregates after 4 h of incubation (Fig. 5B). These results may suggest that no further NETosis occurs in the continuously cultured dHL-60 cells in reductive state since NETosis requires a highly oxidative state in the cells. The reductive/oxidative ratio was compared at 1-h and 4-h incubations of dHL-60 and MSU (Fig. 5C).

### Expression of the positive and negative intracellular cytokine signaling regulators in dHL-60 cells after 2 h and 4 h of interaction with MSU

**Fig. 6:**
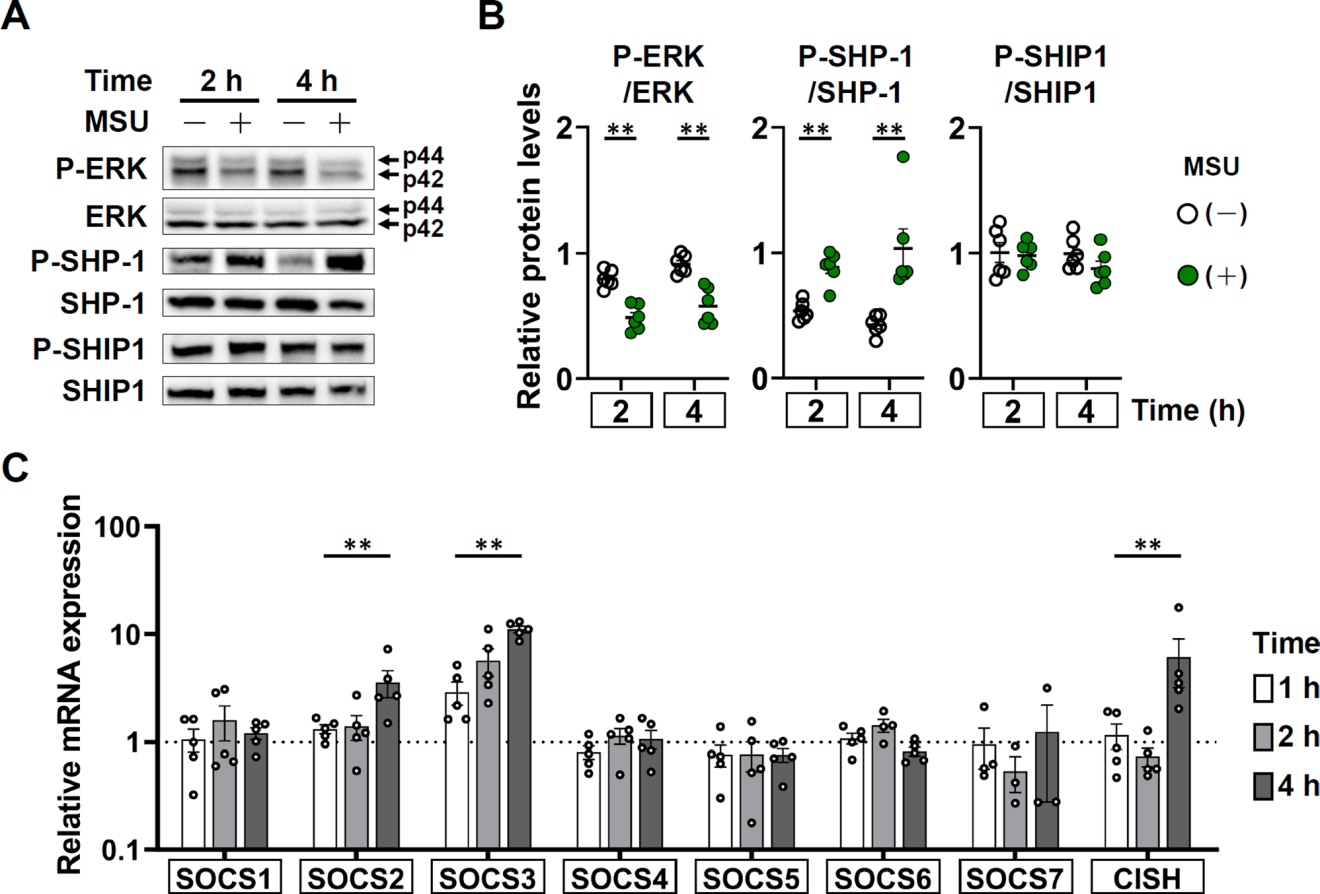
Comparison of positive and negative intracellular cytokine signaling regulators expression after incubation ± MSU. (**A**) A representative case showing the protein expression of P-ERK/ERK, P-SHP-1/SHP-1 and P-SHIP1/SHIP1 intracellular cytokine signaling regulators detected by western blot after 2 h and 4 h incubation. Full membranes are found in supplementary Figure S2. (**B**) Statistical analysis of the relative protein levels of P-ERK/ERK, P-SHP-1/SHP-1, and P-SHIP/SHIP between dHL-60 ± MSU groups after 2 h and 4 h incubation. (**C**) Comparisons of relative mRNA expression of SOCS1-SOCS7 and CISH after incubation of dHL-60 with MSU for 1 h, 2 h and 4 h. Data are shown as mean ± SEM of 5–6 experiments. **p < 0.01 by Mann-Whitney U test

Western blot analysis showed a decreased P-ERK expression (Fig. 6A) and P-ERK/ERK ratio after 2-h interaction (Fig. 6B). Conversely, the P-SHP-1 expression and P-SHP-1/SHP-1 ratio elevated after 2-h incubation with MSU crystals (Fig. 6A and B). However, the P-SHIP-1 expression and P-SHIP1/SHIP1 ratio remained unaffected after 4 h interaction (Fig. 6A and B). In addition, the mRNA expression of the intracellular negative cytokine regulatory molecules SOCS2, SOCS3, and CISH are significantly elevated in 4 h interaction (Fig. 6C). These findings may imply that the MSU stimulated dHL-60 cells gradually shifted the cytokine profile from pro-inflammatory to anti-inflammatory in parallel with NET-MSU aggregate formation.

### N1 to N2 phenotype polarization after interaction of dHL-60 with MSU crystals

**Fig. 7:**
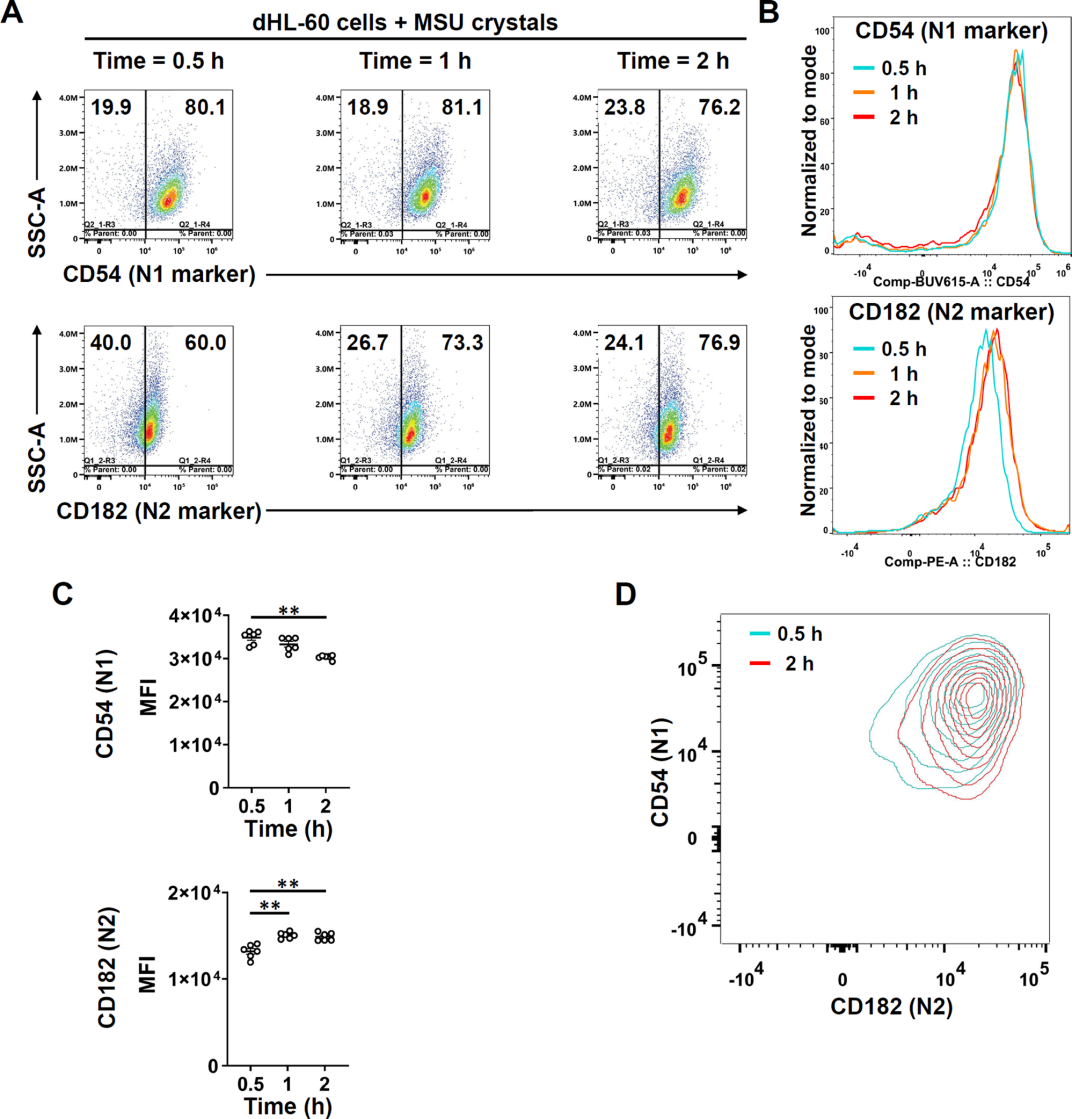
Comparison of surface expressed CD54 (N1) and CD182 (N2) after incubation with MSU. (**A**) Kinetic expression of N1 (upper panel) and N2 (lower panel) biomarkers on dHL-60 cells after incubation with MSU for 0.5 h, 1 h, and 2 h. (**B**) The shift of N1 (upper panel) and N2 (lower panel) expression after incubation with MSU for 0.5, 1, and 2 h. (**C**) Statistical analysis showed significant decrease of N1 (upper panel) but increase of N2 (lower panel) surface marker after 0.5–2 h incubation. (**D**) A representative contour diagram illustrates a polarization trend from CD54^high^CD182^low^ to CD54^low^CD182^high^ cells. Data are shown as mean ± SEM of 6 experiments. **p < 0.01 by Mann-Whitney U test

We next assessed N1 to N2 phenotype polarization of dHL-60 cells after interaction with MSU for 0.5, 1, and 2 h by stain with CD54 (N1 marker) and CD182 (N2 marker) surface expression on dHL-60 cells. A shift from N1 to N2 phenotype was observed along with NET-MSU aggregate formation as detected by flow cytometer (Fig. 7A). The graphic curve is shown in Fig. 7B and statistical analysis is demonstrated in Fig. 7C. In another experiments, only the 2 h culture supernatants obtained from dHL-60 + MSU failed to facilitate N1 to N2 polarization (data not shown). However, the contour diagram clearly demonstrates a gradually shift of CD54^high^CD182^low^ to CD54^low^CD182^high^ phenotype as shown in Fig. 7D. These results may indicate that direct interaction between dHL-60 cells and NET-MSU aggregates is necessary for N1 to N2 polarization.

## Discussion

Previous studies, including our own, have emphasized the role of monocytes/macrophages in resolving acute gout [12–14]. Despite the granulocytes are the most abundant cells in the synovial fluid during acute gout attack, these cells remain a big challenge for investigation because of their short lifespan. However, neutrophils have a short lifespan, with gradually polarization toward N1- or N2-like phenotypes occurring less than 24 h [24]. In this cell-based study, we utilized dHL-60 cells as surrogate granulocytes to investigate the molecular mechanisms underlying the spontaneous resolution of acute gout inflammation.

Recent reports have shown that NETosis could resolve gout flares via degradation of inflammatory cytokines/chemokines by DNA-bound proteolytic enzymes [19, 20]. Furthermore, Garcia-Gonzalez et al. [21] demonstrated that NET release during an acute gout episode depends much more on the amount of MSU crystals rather than the number of infiltrating leukocytes. The concept of “non-inflammatory NETosis” has been also proposed for explaining acute gout resolution through physical sequestration [22, 23]. However, the exact underlying molecular mechanisms for this hypothesis require further confirmation. Our study has unveiled at least the following 5 original observations after prolong incubation of dHL-60 and MSU for 22 h:

1. Concomitant production of pro-inflammatory IL-8 and TNF-α, and anti-inflammatory IL-1RA cytokines during NET-MSU aggregate formation in 4 h incubation.
2. A gradual shift of intracellular cytokine positive signaling regulator P-ERK expression towards negative regulators including CIS, SOCS2 & 3, and P-SHP-1 expression in 4 h interaction.
3. The cellular redox shifted to reductive state in dHL-60 cells for preventing further oxidative-induced NET formation after 4 h of interaction.
4. Gradual shift of dHL-60 cells from pro-inflammatory N1 to anti-inflammatory N2 phenotype when newly added dHL-60 cells encounter with huge NET-MSU aggregates after 1/2 h of interaction.
5. The EIS decreased from a high inflammation of 11.6 at the end of 4 h interaction gradually dropped to 1.53 at the end of the 22-h interaction in that both new MSU crystals and new dHL-60 cells were added mimicking a real clinical scenario after an acute gout episode.

These original findings may potentially explain the spontaneous resolution of acute inflammation induced by MSU, such as in acute gout.

Our findings are quite consistent with the report of Roberge et al. [34] that TNF-α and microcrystals can trigger both neutrophil activation and IL-1RA production. It is conceivable that IL-1RA, one of the IL-1 superfamily, can bind to the IL-1 receptor but fails to trigger IL-1-mediated signaling [35, 36]. Our results are consistent with the notion that granulocyte activation by MSU can simultaneously release both pro-inflammatory (IL-8, TNF-α) and anti-inflammatory (IL-1RA) cytokines in acute gout attack. Initially, IL-1RA levels were insufficient to counteract the inflammatory effects in 4 h incubation, resulting in a high EIS of 11.6 in the acute stage. Then, the intracellular regulatory mechanisms gradually activate the anti-inflammatory mechanisms including shift of cellular redox toward reductive state, enhanced expression of intracellular cytokine negative regulatory signaling molecules, increased IL-1RA production, and shift of N1 to N2 phenotype polarization. These changes are able to mitigate the acute gout inflammation in the later stage in response to large NET-MSU aggregate formation.

Recently, PMNs are categorized into pro-inflammatory N1 and anti-inflammatory N2 phenotypes. Ma et al. [37] firstly demonstrated that neutrophils exhibit temporal polarization after myocardial infarction. Functionally, Ohms et al. [24] identified the N1 phenotype by its elevated ICAM-1 expression, high IFN-γ-induced IP-10/CXCL10 and TNF-α secretion, increased ROS, MPO, and MMP-9 activities, enhanced chemotaxis, and up-regulated ERK and p65NF-κB for mediating acute inflammation. These N1 cells are compatible with our dHL-60 response to MSU within 4 h of incubation. Conversely, Mihaila et al. [38] showed the anti-inflammatory role of N2 phenotype by expressing lower CCL2, CCL3, and CCL5 levels, and reduced MPO and MMP-9 activities in the presence of IL-4. Our unique findings demonstrated that NET-MSU aggregate-induced N2 polarization depends on the direct interaction of N1 cells with NET-MSU aggregates, but not the supernatants obtained from the mixture. In addition, the frustrated N1 cells in facing large NET-MSU aggregates conversely produce much more IL-1RA and less TNF-α to suppress EIS. It is quite interesting that the disruption the DNA scaffold structure in NET-MSU aggregates with DNase I significantly increased the EIS (Fig. 3C). This increase in inflammation is attributed to the fact that small DNA fragments, digested by DNase I, become danger molecules capable of binding to pattern recognition receptors on dHL-60 to induce acute inflammatory responses [33, 39, 40].

**Fig. 8:**
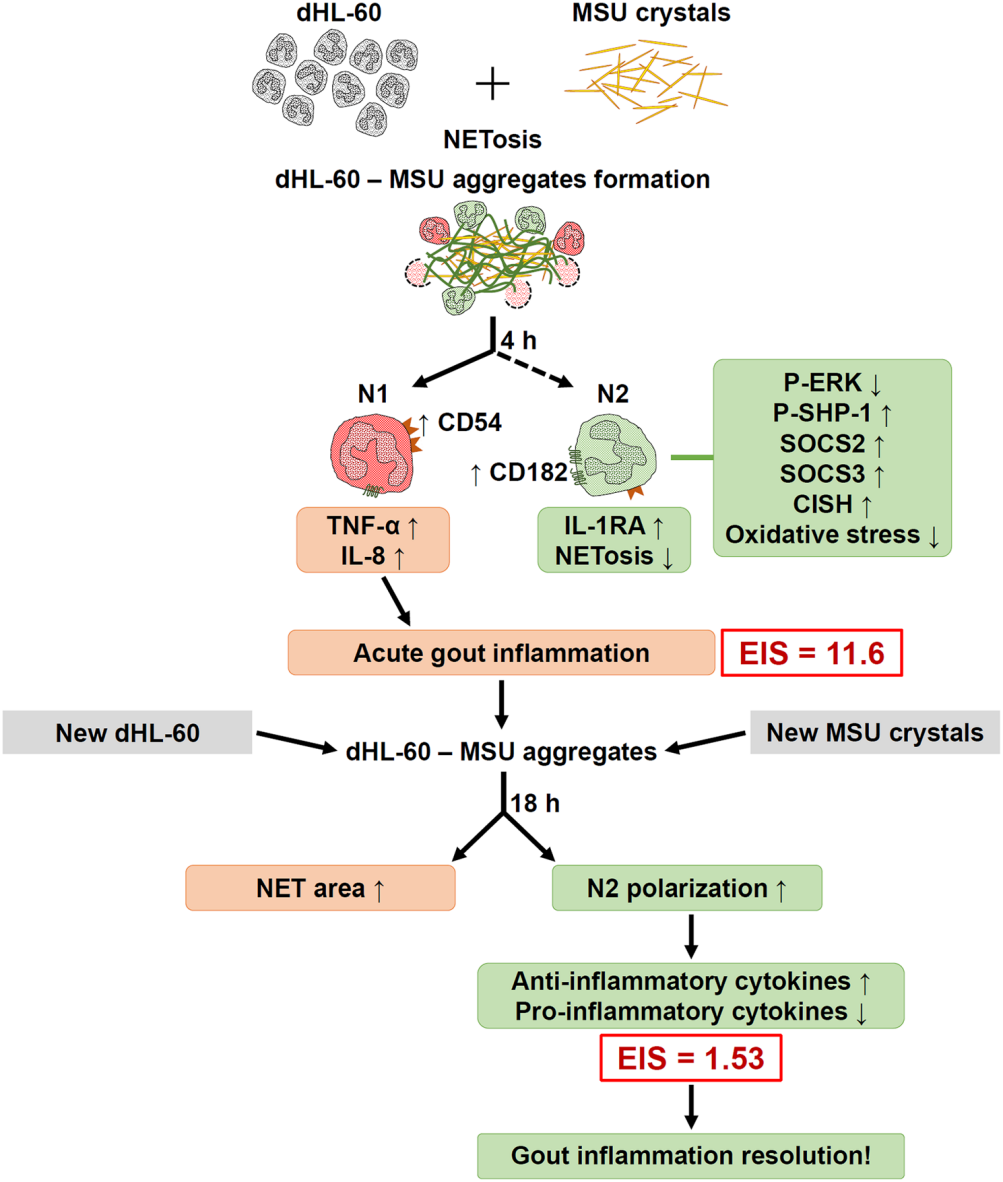
Molecular basis of dHL-60–MSU aggregate in mediating acute inflammation induced by MSU and then spontaneous resolution. MSU activate dHL-60 NETosis accompanied by much more production of pro-inflammatory cytokines (EIS = 11.6) at the end of the 1st reaction. However, the NET-MSU aggregates also gradually activate anti-inflammatory mechanisms including increased IL-1RA production, reduced intracellular cytokine positive signal ERK, decrease oxidative stress, increased intracellular cytokine negative signaling molecules (SOCS2, SOCS3, CISH and SHP-1 phosphorylation) expression, and increased N2 polarization. These mechanisms may facilitate the resolution of MSU-mediated acute inflammation

The intracellular events triggered by NET-MSU aggregates in N2 phenotype include decreased expression of P-ERK, increased expression of SOCS2, SOCS3, CISH, and P-SHP-1 and reduced intracellular ROS production. Moreover, the huge NET-MSU aggregates changed the redox-oxidative active red-colored dHL-60 cells to redox-reductive purple-colored non-NETotic dHL-60 cells around the aggregates (Fig. 5B). These redox-reductive cells fail to underlying NETosis [41]. Instead, these redox-reduced dHL-60 cells can form a barrier for preventing inflammatory cells approaching MSU crystals and increase production of the anti-inflammatory IL-1RA. A graphic summary is shown in Fig. 8.

In conclusion, MSU crystals not only trigger acute inflammation but also stimulate the formation of dHL-60 NET-MSU aggregates in the early stage. Concomitantly, many intracellular anti-inflammatory mechanisms are initiated in these activated dHL-60. Furthermore, sequestration by large NET-MSU aggregates not only frustrates newly entering dHL-60 cells, inducing a transformation from pro-inflammatory N1-like to anti-inflammatory N2-like phenotypes, but also reduces TNF-α and increases IL-1RA production. These cellular changes in dHL-60 cells can lead to the spontaneous resolution of acute inflammation. However, these in vitro cell-based investigations should be further confirmed in vivo.

## Electronic supplementary material

Below is the link to the electronic supplementary material.

Supplementary Material 1

Supplementary Material 2

Supplementary Material 3

## Data Availability

No datasets were generated or analysed during the current study.

## Abbreviations

GA: Gouty arthritis
MSU: Monosodium urate crystals
TGF-β1: Transforming growth factor β1
IL-10: Interleukin-10
TNF-α: Tumor necrosis factor alpha
IL-1RA: IL-1 receptor antagonist
PMN: Polymorphonuclear neutrophils
NETs: Neutrophil extracellular traps
ATRA: All-trans retinoic acid
G-CSF: Granulocyte colony-stimulating factor
dHL-60: Differentiated HL-60 cells
FBS: Fetal bovine serum
CPLM: Compensatory polarized light microscopy
DAPI: 4’,6-diamidino-2-phenylindole
NE: Neutrophil elastase
CitH3: Citrullinated histone H3
IL-8: Interleukin-8
EIS: Estimate Inflammation Score
DNase I: Deoxyribonuclease I
Redox: Oxidation-reduction
FRET: Förster resonance energy transfer
RIPA: Radio-immunoprecipitation assay
ERK: Extracellular signal-regulated kinase
SHP-1: Src homology region 2 domain-containing phosphatase-1
SHIP1: Src homology 2 domain containing inositol polyphosphate 5-phosphatase 1
CISH: Cytokine induced SH2 domain protein
SOCS: Suppressor of cytokine signaling
SEM: Standard error of the mean
