# Supplementary material for: Resolution of acute inflammation induced by monosodium urate crystals (MSU) through neutrophil extracellular trap-MSU aggregate-mediated negative signaling
Source: J Inflamm (Lond). 2024 Nov 27;21:50. doi: 10.1186/s12950-024-00423-9 (PMC11604016; doi:10.1186/s12950-024-00423-9)
Supplement: Supplementary file 2 — Supplementary Material 2 [file 12950_2024_423_MOESM2_ESM.docx]

**Supplementary Video S1.**

The time-lapse observation of fluorescence microscopy images illustrated the formation of NET-MSU aggregates from 0 to 8 hours. The top left images show phase contrast, the top right images reveal extracellular DNA stained with SYTOX Green, the bottom left images represent the live-cell cytoplasm stained with CellTracker™ Red CMTPX Dye, and the bottom right images are the merging photos. Bars = 100 μM.
