# Supplementary material for: Resolution of acute inflammation induced by monosodium urate crystals (MSU) through neutrophil extracellular trap-MSU aggregate-mediated negative signaling
Source: J Inflamm (Lond). 2024 Nov 27;21:50. doi: 10.1186/s12950-024-00423-9 (PMC11604016; doi:10.1186/s12950-024-00423-9)
Supplement: Supplementary file 3 — Supplementary Material 3 [file 12950_2024_423_MOESM3_ESM.docx]

**Supplementary Figure Titles and Legends**

**Figure S1. The purity of CD11b-expressing cells.**

**
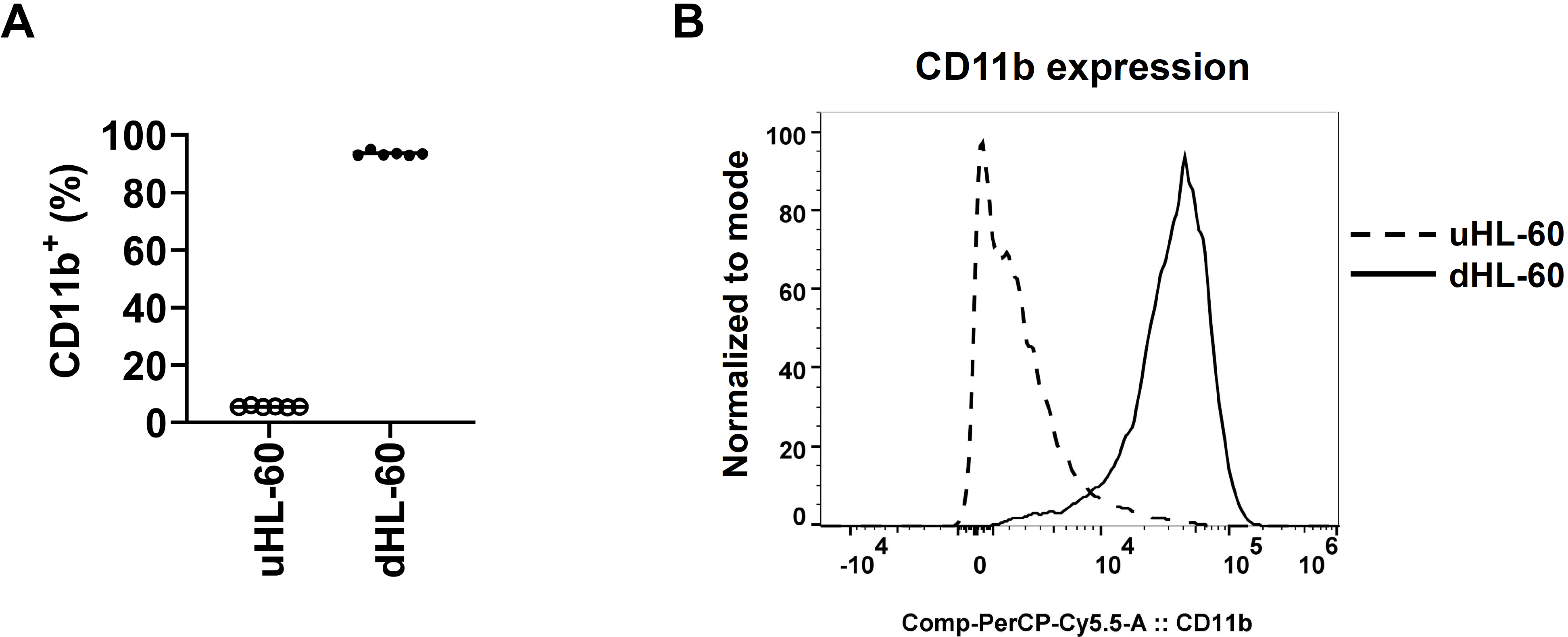
**

**(A)** The expression of CD11b in undifferentiated HL-60 (uHL-60) is low. After differentiation induced by all-trans-retinoic acid, more than 90% of the differentiated HL-60 (dHL-60) express CD11b. **(B)** A representative flow cytometry plot showing CD11b expression in uHL-60 and dHL-60 cells. Data are shown as mean ± SEM of 6 experiments.

**Figure S2.** **Unedited western blot images for Figure 6A.**

**
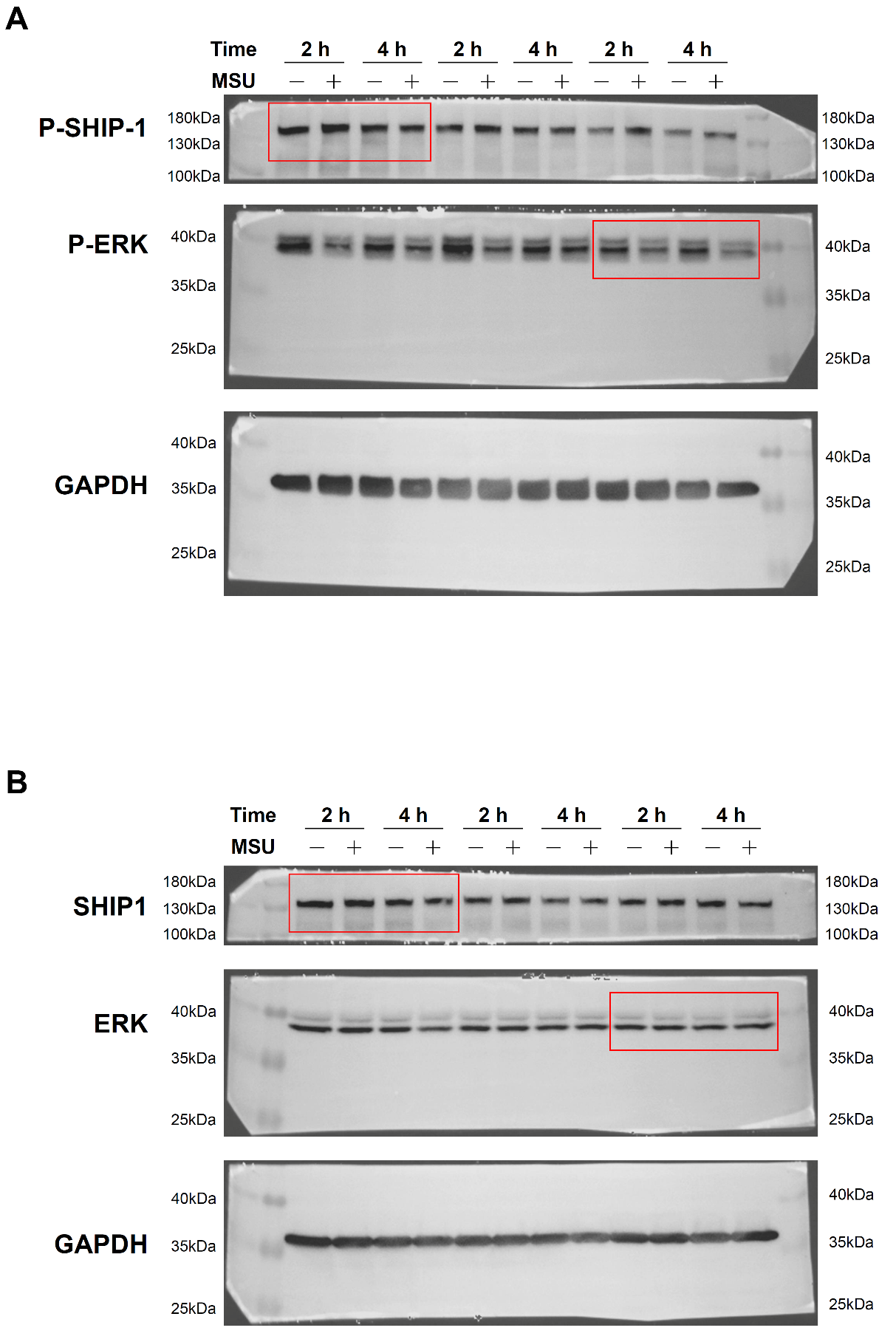
**

**
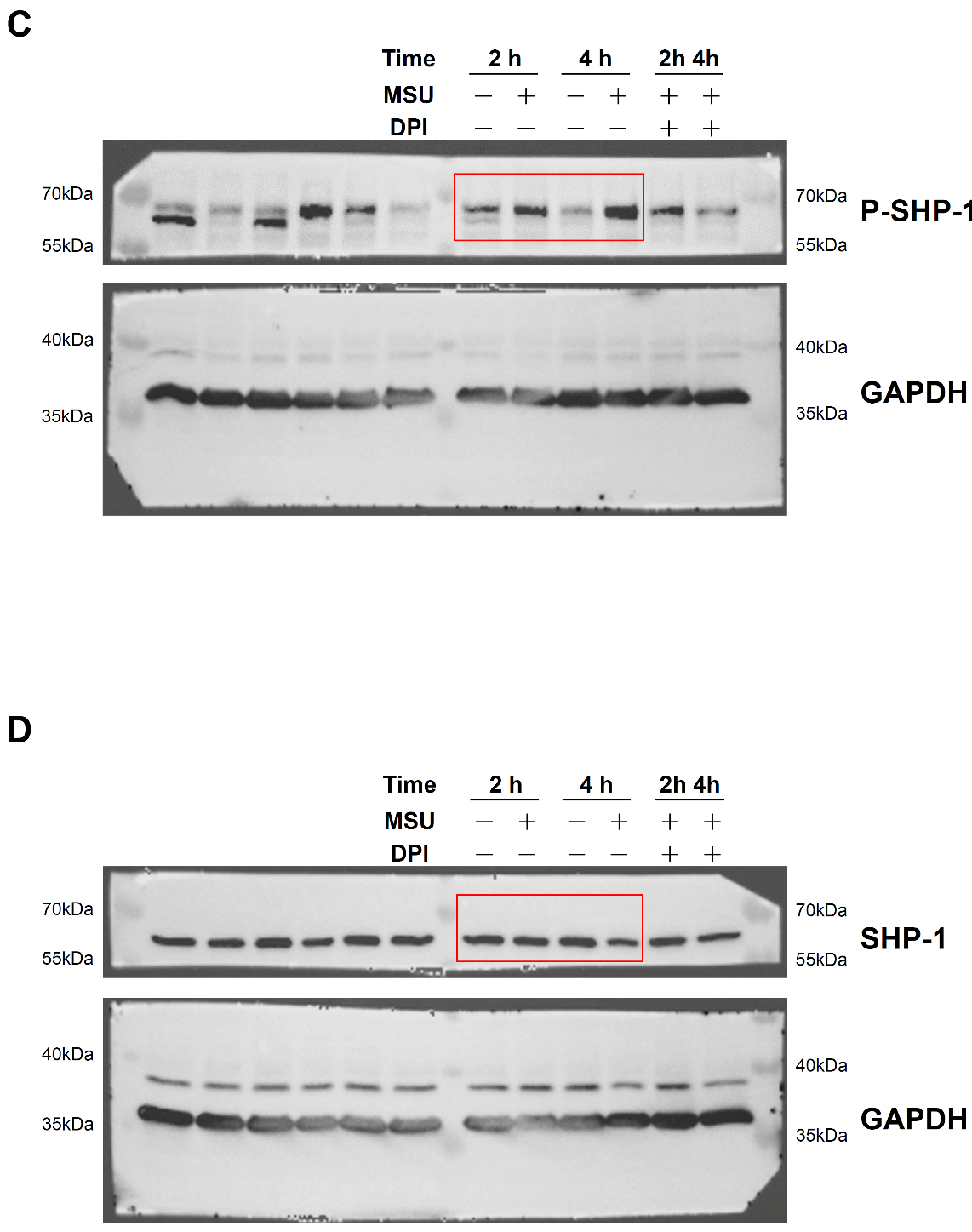
**

**Supplementary Table**

**Table S1.** The list of primer pairs for SOCS1–SOCS7, CISH and cyclophilin A.

| **Gene** | **Forward** | **Reverse** |
| --- | --- | --- |
| SOCS1 | TTCGCCCTTAGCGTGAAGATGG | TAGTGCTCCAGCAGCTCGAAGA |
| SOCS2 | CAGATGTGCAAGGATAAGCGG | GCGGTTTGGTCAGATAAAGGTG |
| SOCS3 | CATCTCTGTCGGAAGACCGTCA | GCATCGTACTGGTCCAGGAACT |
| SOCS4 | GGGTAAGCACAGACTTGTCTCAG | TCACAGAGCCAGTCATAGGACC |
| SOCS5 | TCTGGAGACAGCCATACCCATG | GCTTCATAACGGTCCATCACTCC |
| SOCS6 | CGAGGATGAGAGTCAGGTAGAC | GGTAGCAATGGTGAGAGTGGAG |
| SOCS7 | ACAGGTGAAACTGTGTCGCTT | TAGGCAGCGTCCCACTGATA |
| CISH | TGCCAGAAGGCACGTTCTTAG | GCCACGAGTGGTTTTCACTG |
| Cyclophilin A | GCATACGGGTCCTGGCATCTTGTCC | ATGGTGATCTTCTTGCTGGTCTTGC |
